# Asymptotic dynamics of some *t*-periodic one-dimensional model with application to prostate cancer immunotherapy

**DOI:** 10.1007/s00285-016-0978-4

**Published:** 2016-02-20

**Authors:** U. Foryś, M. Bodnar, Y. Kogan

**Affiliations:** 1 Faculty of Mathematics, Informatics and Mechanics, Institute of Applied Mathematics and Mechanics, Warsaw University, Warsaw, Poland; 2 Institute for Medical BioMathematics, Bene Ataroth, Israel

**Keywords:** Prostate cancer, Immunotherapy, Mathematical model, 34A37, 34C25, 34C60, 37N25, 92B05

## Abstract

In the case of some specific cancers, immunotherapy is one of the possible treatments that can be considered. Our study is based on a mathematical model of patient-specific immunotherapy proposed in Kronik et al. (PLoS One 5(12):e15,482, 2010). This model was validated for clinical trials presented in Michael et al. (Clin Cancer Res 11(12):4469–4478, 2005). It consists of seven ordinary differential equations and its asymptotic dynamics can be described by some *t*-periodic one-dimensional dynamical system. In this paper we propose a generalised version of this *t*-periodic system and study the dynamics of the proposed model. We show that there are three possible types of the model behaviour: the solution either converges to zero, or diverges to infinity, or it is periodic. Moreover, the periodic solution is unique, and it divides the phase space into two sub-regions. The general results are applied to the PC specific case, which allow to derive conditions guaranteeing successful as well as unsuccessful treatment. The results indicate that a single vaccination is not sufficient to cure the cancer.

## Introduction

Over the last few decades, cancer immunotherapy has become a growing area of active research, with a wide variety of approaches developed and clinically implemented in different cancer indications. The rational basis for immunotherapy is the assumption that cancer evolves to evade the control of the host’s immune system, and that a proper boost, e.g. by restoration of an impaired function or by countering immunosuppressive mechanisms employed by the cancer, would allow immune system to regain control over the disease (de Visser et al. 2006; Hanahan and Weinberg 2011). Thus, the common denominator of the different modes of cancer immunotherapy is the attempt to manipulate one or several components of patient’s own immune system that interact with the disease (in contrast to the chemical or biological therapies that directly target the cancer or cancer-supporting cells). This adds further levels of complexity to the system, making understanding and rationalisation of the treatment effects a non-trivial task.

In order to effectively respond to a developing malignancy, the immune system has to properly recognise the threat, induce massive production of several types of immune cells that perform different complementary tasks, create the suitable micro-environment and successfully deliver the relevant cells and substances to the disease location. Cancer evades the immune control by acquiring the ability to impede these processes at one or more stages, e.g., by reducing the efficacy of antigen recognition, or suppressing the immune response by secreting anti-inflammatory or pro-regulatory cytokines. Many different ways in which cancer may protect itself from the immune system have been discovered (Dunn et al. 2002; Schreiber et al. 2011). When these evasion strategies are understood, the immunotherapy approaches can be designed in order to overcome them by enhancing the impaired immune processes. The variety of suggested immune treatments includes therapeutic vaccines, cytokines, and monoclonal antibodies enhancing tumor-killing mechanisms of the adaptive immune response (Gulley and Drake 2011; Melero et al. 2014). Each treatment type targets a specific component of the complicated network of interactions between the immune system and cancer. Yet, the suggested treatments encounter significant problems when translated into clinic, eventually showing limited efficacy and low response rate, even though sporadic cases of remarkable effectiveness are observed in individual patients. The latter observation raises the possibility that effective design of immune intervention is undermined by our limited understanding of the complicated dynamics of interactions between the two major players, the immune system and the cancer, and how it varies from patient to patient (Agur and Vuk Pavlovic’ 2012).

Mathematical analysis is a powerful way to gain understanding of behaviour of complex systems, in particular, in biology. Indeed, cancer–immune interactions have been extensively investigated using various mathematical models; see recent reviews in de Pillis et al. (2014) and Eftimie et al. (2011) and references therein. One popular approach is to represent the studied system by a set of differential equations, whereas the time-dependent variables represent the quantities of cells or substances of interest. The equations’ structure reflects the understanding of the most important processes and interactions, whereas the parameters represent the rates or relations that characterise these processes. Such a model can be then investigated analytically or numerically (by simulations) in order to determine the spectrum of plausible behaviours and, if possible, gain insight into optimal design of therapeutic intervention. Hitherto, many different models have been formulated and studied, most of them emphasising the theoretical aspects of the immunotherapy, with limited application to actual experimental and clinical data. Yet, if the model’s parameters are retrieved from experiments, or by matching model output versus real clinical data, one can obtain not only a general insight into the systems’ dynamics, but rather actually predict the quantitative response of the disease to the treatment. Further, this allows designing and testing various alternative treatment strategies that would be most beneficial, on a population or individual level. Following this line of thought, a model of progression of prostate cancer (PCa) under vaccination treatment was developed and calibrated in Kronik et al. (2010), using data published in the literature, as well as individual profiles of prostate-specific antigen (PSA) in response to vaccination by allogeneic whole-cell vaccination in a phase 2 clinical study (Michael et al. 2005). The model, comprising a system of 7 ordinary differential equations (ODEs), was calibrated individually for each patient, by fitting the individual response data, given as PSA levels measured before, during and after the treatment. It was able to accurately reproduce individual PSA dynamics in response to immunotherapy, for most of the patients. In a subsequent work, it was also demonstrated that individual parameters can be identified at early stages of the treatment, and thus, the model can be used to predict further dynamics of response and eventually optimise the individual treatment schedule, given a well-defined end-point; e.g., stabilisation of PSA levels (Kogan et al. 2012). Thus, such a model may be relevant to a clinical application of immunotherapy, with a potential to dramatically change the approach to treatment design. All the above investigation was carried by numerical simulations, which has the obvious drawback of exhibiting model behaviour only under the specific sets of parameters values, at which the model is numerically solved. Here, we wish to expand this investigation by an analytical study of the asymptotic behaviour of this specific model. This will allow further insight into its predictive ability, scope and limits of applications, which can be important to further utilisation of the model, and the above approach as a whole.

This article is built as follows. Section 2 presents a general result concerning asymptotic dynamics of one ODE with the right hand-side *F*(*t*, *x*) being *t*-periodic and monotonic in *x*. Section 3 briefly presents and explains the model to be studied. Section 4 shows that the system dynamics is asymptotically one dimensional and particularly simple when only one boost of immunotherapy is given. Finally, Sect. 5 presents the analysis for the case of periodic impulsive vaccination. In that case giving the treatment periodically, we asymptotically obtain a *t*-periodic right-hand side of the equation, and the general theorem from Sect. 2 applies.

## General theorems

We consider a general Cauchy problem of the form

1 $$\begin{aligned} \dot{x} = x \, F(t,x), \quad x(t_0)=x_0, \quad x_0,\ t_0\ge 0, \end{aligned}$$

where the function *F* fulfils the following conditions.

1. *F* is continuous and locally Lipschitz continuous in *x* for all $(t,x) \in \mathscr {D}= {\mathbb {R}}_+^2$, where ${\mathbb {R}}_+ =[0,+\infty )$.
2. For all $(t,x)\in \mathscr {D}$ equality $F(t+1,x)=F(t,x)$ holds, that is *F* is *t*-periodic.
3. *F* is increasing in *x*, that is for any $t\ge 0$ and $x>y\ge 0$ inequality $F(t,x)>F(t,y)$ holds.
4. *F* is uniformly bounded in $\mathscr {D}$.

### *Remark 1*

Notice that due to *t*-periodicity of *F* it is enough to consider initial time $t_0\in [0,1)$. Moreover, Assumptions (A1)–(A4) imply that unique solutions of Eq. (1) exist globally in time (that is for every $t \ge 0$) independently of $t_0\in [0,1)$ and $x_0 \ge 0$. Hence, for $x_0>0$ there is $x(t)>0$ for any $t \ge 0$.

### **Theorem 1**

Let *F* fulfil (A1)–(A4) and

$$\begin{aligned} F_A~= \int \limits _0^1 F(s,0)\,ds>0. \end{aligned}$$

Then any solution of Eq. (1) with $x_0>0$ tends to $+\infty$ as $t\rightarrow +\infty$.

### *Proof*

Let $t \in [0,1)$ and $n\in {\mathbb {N}}$ be arbitrary. Integrating Eq. (1) on the interval $[t+n,t+n+1]$ we obtain

$$\begin{aligned} \ln \frac{ x(t+n+1)}{x(t+n)} = \int \limits _{t+n}^{t+n+1} F(s,x(s)) ds, \end{aligned}$$

and therefore monotonicity of *F* in *x* implies

$$\begin{aligned} \begin{aligned} x(t+n+1)&= x(t+n) \exp \left( \int \limits _{t+n}^{t+n+1} F(s,x(s))ds \right) \\&> x(t+n) \exp \left( \int \limits _{t+n}^{t+n+1} F(s,0)ds \right) =x(t+n)\text {e}^{F_A}. \end{aligned} \end{aligned}$$

Under the assumption $F_A>0$ the sequence $( x(t+n))_{n\in {\mathbb {N}}}$ satisfies the inequality $x(t+n+1)>q x(t+n)$ with $q=\text {e}^{F_A}>1$. As $t \in [0,1)$ is arbitrary, this yields $x(t)\rightarrow +\infty$ as $t\rightarrow +\infty$. $\square$

### **Theorem 2**

Let *F* fulfil (A1)–(A4) and

$$\begin{aligned} F_A~= \int \limits _0^1 F(s,0)\,ds<0. \end{aligned}$$

Moreover, assume that

$$\begin{aligned} F(t,x)\rightarrow f(t)>0 \quad \text {uniformly as} \ x\rightarrow +\infty . \end{aligned}$$

Then there exists a curve $\gamma : [0,1)\rightarrow (0,+\infty )$ such that

- if $x_0<\gamma (t_0)$, then solutions of Eq. (1) converge to zero;
- if $x_0>\gamma (t_0)$, then solutions of Eq. (1) diverge to $+\infty$;
- if $x_0=\gamma (t_0)$, then $x(t_0+1)=\gamma (t_0)$ and the curve $\gamma$ prolonged in a periodic way on $[1,+\infty )$ is a periodic solution of Eq. (1).

### *Proof*

**Fig. 1:**
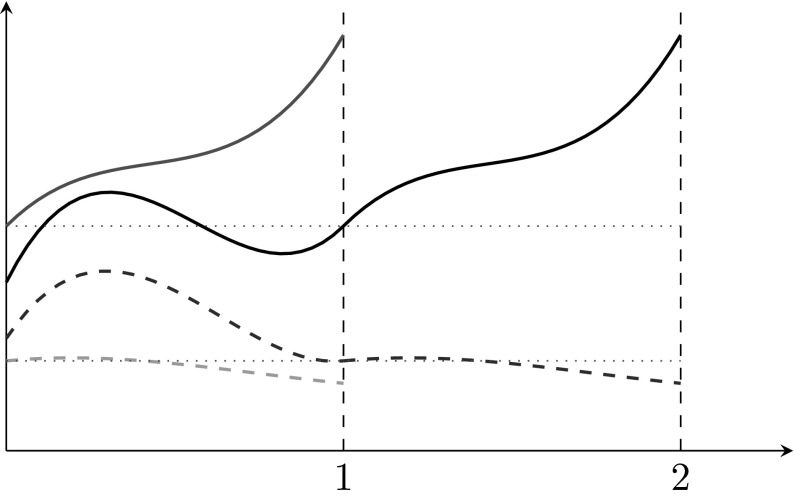
Illustration of the behaviour of solutions of Eq. (1)

According to Remark 1 solutions of Eq. (1) are unique, and thus trajectories cannot cross each other. We show that the behaviour of the solution is thus determined by the inequality between values at $t=t_0$ and $t=t_0+1$, cf. Fig. 1 for $t_0=0$.

If $x_0=x(t_0)<x(t_0+1)=x_1$, then this inequality is preserved for any $t \ge 0$. Clearly, considering the trajectory *x*(*t*) for initial data $(t_0,x_0)$ it lies below the trajectory $\tilde{x}(t)$ for initial data $(t_0,x_1)$, as solutions are unique. However, $\tilde{x}(t)$ on $[t_0,t_0+1]$ is a shift of *x*(*t*) from $[t_0+1,t_0+2]$ due to *t*-periodicity of the vector-field *F*. Similarly, if $x(t_0)>x(t_0+1)$, then $x(t)>x(t+1)$ for any $t \ge 0$.

Consider the case $x(t_0)<x(t_0+1)$. Due to Assumption (A3) we have

$$\begin{aligned} \int \limits _{t_0+n-1}^{t_0+n} F(s,x(s))ds > \int \limits _{t_0}^{t_0+1} F(s,x(s))ds \end{aligned}$$

for any $n>1$. Notice, that $f_A(x)=\int _{t_0}^{t_0+1}F(t,x)dt$ is a continuous, increasing function of *x*. Moreover $f_A(0)=F_A<0$ and $f_A(\infty )>0$ according to the assumptions. Therefore, there exists $\bar{x}>0$ such that $f_A(x)<0$ for $x<\bar{x}$, and $f_A(x)>0$ for $x>\bar{x}$. Therefore, for $x_0 \ge \bar{x}$ we have

$$\begin{aligned} \delta (t_0)= \int \limits _{t_0}^{t_0+1} F(s,x(s))ds >\int \limits _{t_0}^{t_0+1} F(s,x_0)ds \ge 0, \end{aligned}$$

which yields

$$\begin{aligned} x(t_0+n) \ge x_0 \text {e}^{\delta (t_0) n} \rightarrow +\infty \end{aligned}$$

for any $$t_0\in [0,1)$$.

For the case $\bar{x}>x(t_0)>x(t_0+1)$ analogous arguments leads to the following conclusion

$$\begin{aligned} \begin{aligned} x(t_0+n)&\le x_0 \exp \left( n \int \limits _{t_0}^{t_0+1} F(s,x(s))ds\right) \\&< x_0 \exp \left( n \int \limits _{t_0}^{t_0+1} F(s,x_0)ds\right) = x_0\text {e}^{n\delta (t_0)} \rightarrow 0 \end{aligned} \end{aligned}$$

for any $t_0\in [0,1)$, as $\delta (t_0)<0$ in this case.

It remains to prove that a periodic solution exists. We show that if $x_0$ is small enough, then $x(t)\rightarrow 0$, while if $x_0$ is large enough, then the solution diverges to $+\infty$. The continuous dependance of solutions on initial data would complete the proof.

To this end, first we give some estimates of the solutions. Due to (A4) there exists $r>0$ such that $|F(t,x)|<r$ for all $(t,x)\in \mathscr {D}$. Thus, for any $t \in [t_0, t_0+1)$ we have

$$\begin{aligned} x(t)=x_0 \exp \left( \int \limits _{t_0}^t F(s,x(s)) ds \right) \le x_0 \text {e}^r \end{aligned}$$

and,

$$\begin{aligned} x(t)\ge x_0 \exp \left( \int \limits _{t_0}^t F(s,0) ds \right) \ge x_0 \text {e}^{-r}. \end{aligned}$$

Now, consider $x_0$ sufficiently small. Then

$$\begin{aligned} x(t_0+1)=x_0\exp \left( \int \limits _{t_0}^{t_{0}+1}F(s,x(s))ds \right) \le x_0\exp \left( \int \limits _{t_0}^{t_{0}+1}F(s,x_0\text {e}^r)ds \right) . \end{aligned}$$

Let $\bar{x}$ be as before, that is $f_A~(x) <0$ for all $0\le x< \bar{x}$. For $x_0\le \bar{x} \text {e}^{-r}$ we have

$$\begin{aligned} \exp \left( \int \limits _{t_0}^{t_0+1}F(s,x(s))ds \right) < \exp \left( \int \limits _{t_0}^{t_0+1}F(s,\bar{x})ds\right) <1, \end{aligned}$$

and therefore $$x(t_0+1)<x(t_0)$$.

Similarly, we can show that for $x_0$ sufficiently large $x(t_0+1)> x(t_0)$. Clearly,

$$\begin{aligned} x(t_0+1)> x_0 \exp \left( \int \limits _{t_0}^{t_0+1} F(s,x_0\text {e}^{-r})ds \right) . \end{aligned}$$

As $f(t)=\lim \nolimits _{x\rightarrow \infty } F(t,x)>0$ and due to continuity and *t*-periodicity of *F* the function *f* is continuous periodic and thus there exists $\varepsilon >0$ such that $f(t)>\varepsilon$. The uniform convergence of $F(t,\cdot )$ to *f*(*t*) implies that there exists $\tilde{x}$ such that for all $x\ge \tilde{x}$ the inequality $F(t,x)>\varepsilon /2$ holds. Thus, for $x_0>\tilde{x}$ we have

$$\begin{aligned} \dot{x} \ge \frac{\varepsilon }{2}x \quad \Longrightarrow \quad x(t_0+1) \ge x_0 \text {e}^{\varepsilon t/2} > x(t_0). \end{aligned}$$

Now, continuous dependance and uniqueness of solutions of Eq. (1) finishes the proof.

### *Remark 2*

Notice, that if *F*(*t*, *x*) is independent of *t*, that is $F(t,x)=G(x)$ for some locally Lipschitz continuous function *G*, then

1. either there exists $\tilde{x}>0$ such that $G(\tilde{x})=0$, and then $x(t) \rightarrow 0$ for $0<x_0<\tilde{x}$, while for $x_0>\tilde{x}$ we have $x(t) \rightarrow +\infty$ as $t \rightarrow +\infty$;
2. or $G(x)>0$ for $x>0$, and then $x(t) \rightarrow +\infty$ for any $x_0>0$.

In the next section we use our results to analyse the PC immunotherapy model proposed in Kronik et al. (2010).

## A model for PCa immunotherapy

In this section we present and briefly explain the model for vaccination treatment of PCa which was proposed in Kronik et al. (2010). This model describes interactions among the tumour antigen, either endogenous ($V_p$) or injected as a vaccine extracted from cell lines (*V*), several types of immune cells and prostate cancer cells, *P*. The injected vaccine is assumed to be taken up by the naive dendritic cells. It is injected into the dermis where it stimulates maturation of dendritic cells (we assume that there is a large pool of immature dendritic cells) into mature antigen-presenting cells ($D_m$) at the rate $k_i$; cf. Banchereau and Palucka (2005) and Banchereau and Steinman (1998). Each dendritic cell takes up an amount of vaccine, $n_V$, during maturation. The mature antigen-presenting dendritic cells migrate from skin into lymph nodes at the rate $k_m$ with the probability $\alpha _l$ to join the pool of functional antigen-presenting dendritic cells ($D_C$). The functional dendritic cells become exhausted (Kajino et al. 2007) and turn into regulatory dendritic cells ($D_R$) (Langenkamp et al. 2000) at the rate $k_CR$, and the latter die with rate $\mu _D$. Moreover, functional antigen-presenting dendritic cells recruit and activate tumor-specific cytotoxic T lymphocytes (CTLs; *C*) (Janeway et al. 2005) that can kill tumor cells, at the rate $a_C$. In parallel, regulatory dendritic cells recruit regulatory lymphocytes, known as Tregs (*R*) (Hollenbaugh and Dutton 2006), at the rate $a_R$. The Tregs inactivate CTLs (George et al. 2003) with rate proportional to both cell type concentrations, with the coefficient $k_CR$. Both types of lymphocytes, CTLs and Tregs, are also dying with rates $\mu _C$ and $\mu _R$, respectively. The existence of regulatory cells cannot be ignored as increased number of regulatory CD$4^+$CD$25^{\text {high}}$ T cells were detected in the blood and tumour tissue of early stage PCa patients (Miller et al. 2006). Finally, the tumour cells, *P*, are assumed to grow exponentially with rate *g* in absence of CTLs, while the latter kill the tumour cells with rate proportional to both populations, with coefficient $a_P$ and additional factor that decrease tumour killing efficacy with increasing tumor burden (cf. Kronik et al. 2008; Kogan et al. 2010; Piotrowska et al. 2013 for additional information about this saturation). All the variables related to the specific cell populations, reflect amounts of the corresponding cells at time *t*. The above-described cascade of immune reactions that eventually leads to the suppression of cancer cells, is represented by the following system of ordinary differential equations

2 $$\begin{aligned} \begin{aligned} \dot{V}&= -k_l n_V V, \\ \dot{D}_m&= k_l( V+V_p)-k_m D_m, \\ \dot{D}_C&= \alpha _l k_m D_m-k_{CR} D_C, \\ \dot{D}_R&= k_{CR} D_C-\mu _D D_R, \\ \dot{R}&= a_R D_R-\mu _R R, \\ \dot{C}&= a_C D_C-\mu _C C - k_R CR, \\ \dot{P}&= g P - a_P \frac{h_p CP}{h_P+P}, \end{aligned} \end{aligned}$$

with an initial condition reflecting one boost of $V_0$ to the system without immunity, that is $(V_0,0,0,0,0,0,P_0)$, $V_0$, $P_0>0$. Later, we study addition of multiple boosts, which can be represented by adding appropriate $\delta$-functions (i.e. $V_i \cdot \delta (t-t_i)$ for each boost $V_i$ at time $t_i$) to the first equation in (2). For the comparison with previous analysis, note, that in Kogan et al. (2012) and Kronik et al. (2010) it was assumed that $V_p=0$, i.e. the endogenous immune response induced by tumour antigens is either fully suppressed, or negligible. In this case, without treatment, asymptotically, the tumour always will grow without limit, while other system components will stay at zero. All other parameters were estimated (averages and biologically plausible ranges) in Kronik et al. (2010).

For facilitation of our analysis, we non-dimensionalise this system, in order to reduce the number of parameters. New parameter values can be easily computed from the formulae below and the estimations reported in Kronik et al. (2010). We define the following variables and parameters:

$$\begin{aligned} \begin{array}{l} d_m = \dfrac{D_m}{k_l}, \quad d_c = \dfrac{D_C}{\alpha _l k_l k_m}, \quad d_r\! =\! \dfrac{D_R}{\alpha _l k_l k_m k_{CR}}, \quad r \!=\! \dfrac{R}{\alpha _l k_l k_m k_{CR} a_R}, \quad c \!=\! \dfrac{C}{\alpha _l k_l k_m a_C},\\ p = \dfrac{P}{h_p}, \quad k = \alpha _l k_l k_m k_{CR} a_R k_R, \quad k_v = k_l n_V,\quad a~= a_P a_C \alpha _k k_m k_l. \end{array} \end{aligned}$$

These substitutions transform the system into the following form:

3 $$\begin{aligned} \begin{aligned} \dot{V}&= -k_v V, \\ \dot{d}_m&= V+V_p-k_m d_m, \\ \dot{d}_c&= d_m-k_{CR} d_c, \\ \dot{d}_r&= d_c-\mu _D d_r, \\ \dot{r}&= d_r-\mu _R r, \\ \dot{c}&= d_c-\mu _C c - k c r, \\ \dot{p}&= g p - a~\frac{cp}{1+p}. \end{aligned} \end{aligned}$$

The non-dimensional formulation reduces the number of parameters from 14 to 9, without changing the qualitative dynamics. In fact, the original variables values are simple linear functions of those in (3), and can be easily recomputed, once the parameters are given. In the following, we study the behaviour of system (3).

## Dynamics of Eqs. (3) after one boost

Studying the dynamics of Eqs. (3) we can integrate subsequent equations one by one obtaining

$$\begin{aligned} V(t) =V_0\text {e}^{-k_v t} \quad \Longrightarrow \quad V(t) \rightarrow 0 \quad \ \text {as} \ \ t \rightarrow \infty . \end{aligned}$$

Therefore,

$$\begin{aligned} \dot{d}_m = V_0\text {e}^{-k_v t} +V_p -k_m d_m \end{aligned}$$

implying

$$\begin{aligned} d_m = \frac{V_p}{k_m}(1-\text {e}^{-k_m t} ) +\frac{V_0}{k_m-k_v}( \text {e}^{-k_v t}-\text {e}^{-k_m t} ), \end{aligned}$$

as $k_m\ne k_v$ for the parameter values estimated in Kronik et al. (2010). Therefore, $d_m \rightarrow \frac{V_p}{k_m}$, and moreover if $V_p=0$, then $d_m \rightarrow 0$ exponentially.

Next, we can also obtain an explicit formula for $d_c$, and now as $k_{CR}=k_m$ in the original parameter estimation, we have not only exponential functions, but also combinations of exponential functions with a linear term *t*. However, the full expression for $d_c$ is not important for the asymptotic behaviour of the solutions. The first five equations of (3) form a linear subsystem, and it is easily seen that $d_c \rightarrow \frac{V_p}{k_m k_{CR}}$ and again $d_c \rightarrow 0$ for $V_p=0$, and the convergence is of the order $t\text {e}^{-k_{CR}t}$.

Similarly,

$$\begin{aligned}&d_r \rightarrow \frac{V_p}{k_m k_{CR} \mu _D} \quad \Longrightarrow \quad d_r \rightarrow 0 \text {for} \ \ V_p=0, \\&r \rightarrow \frac{V_p}{k_m k_{CR} \mu _D\mu _R} \quad \Longrightarrow \quad r \rightarrow 0 \text {for} V_p=0, \\&c \rightarrow \frac{\mu _D\mu _R V_p}{k_{CR} k_m \mu _C\mu _R\mu _D + kV_p}=: c_{\infty } \Longrightarrow c \rightarrow 0 \text {for} \ \ V_p=0. \end{aligned}$$

Eventually, for every $\varepsilon >0$ there exists $\bar{t}>0$ such that

4 $$\begin{aligned} p\left( g - \frac{a c_{\infty } }{1+p} -\varepsilon \right) \le \dot{p} \le p\left( g - \frac{a c_{\infty } }{1+p} +\varepsilon \right) . \end{aligned}$$

Let us consider one-dimensional dynamical system governed by

5 $$\begin{aligned} \dot{x} = x\left( \tilde{g} - \frac{a c_{\infty } }{1+x} \right) , \end{aligned}$$

where $\tilde{g} = g+\varepsilon$ or $\tilde{g} = g-\varepsilon$. According to Remark 2, the dynamics of Eq. (5) depends on the magnitude of $c_{\infty }$. Clearly,

- If $c_{\infty }$ is small, such that Eq. (5) has no positive equilibrium, then $x \rightarrow \infty$.
- If $c_{\infty }$ is sufficiently large, then there exists a positive equilibrium $\bar{x} = \frac{a c_{\infty }}{\tilde{g}} - 1$ of Eq. (5) and for $0<x_0<\bar{x}$ the solution tends to 0, while for $x_0>\bar{x}$ the solution tends to $\infty$.

As $\varepsilon$ is arbitrary, to have a positive equilibrium $\bar{p}$ one needs

$$\begin{aligned} \bar{p} = \frac{a c_{\infty }}{g} - 1 >0 \ \Longleftrightarrow \ c_{\infty } > \frac{g}{a }. \end{aligned}$$

This condition is equivalent to

$$\begin{aligned} \frac{\mu _D\mu _R V_p}{k_{CR} k_m \mu _C\mu _R\mu _D + kV_p}>\frac{g}{a}, \end{aligned}$$

that is

$$\begin{aligned} V_P \left( a\mu _D\mu _R - g k\right) > g k_m \mu _C \mu _D \mu _R k_{CR}. \end{aligned}$$

### **Corollary 1**

To achieve a cure of the disease one needs

$$\begin{aligned} g< \frac{ a~\mu _D\mu _R}{ k } :=g_{\max } \quad \text {and} \quad V_P > \frac{ g k_m \mu _C \mu _D \mu _R k_{CR}}{k(g_{\max } - g ) }. \end{aligned}$$

Corollary 1 means that to achieve the cure after one boost the tumour cannot be highly reproductive and moreover natural influx $V_P$ of mature dendritic cells must be sufficiently large.

It is obvious that in this case,

- if $$p \in (0, \bar{p})$$, then $$p \rightarrow 0$$;
- if $p>\bar{p}$, then $\dot{p}>0$ and $p \rightarrow \infty$.

Moreover, if $c_{\infty } < \frac{g}{a }$, then there is no positive equilibrium and *p* also tends to $\infty$.

Notice, that if $V_p=0$ we have $c_{\infty }=0$, and therefore for any positive initial data $p(0)=p_0$ we have $p(t) \rightarrow \infty$.

### **Corollary 2**

For parameter values estimated in Kronik et al. (2010) the cure cannot be achieved after one boost.

## Impulsive vaccination

Following Corollary 2, when $V_p=0$ (or when it is very small) we need to apply more vaccination boosts in order to reduce tumour load. In the following we assume $V_p=0$ and consider the sequence of boosts of the same magnitude, $V_0$, given each time interval $\varDelta t$, starting at $t=0$. We obtain impulsive equation for *V* which can be easily solved on intervals of the form $[n\varDelta t,(n+1)\varDelta t]$, $n\in {\mathbb {N}}$. Clearly, for $t \in (n\varDelta t,(n+1)\varDelta t)$ we obtain

$$\begin{aligned} V(t)=V_0 \frac{1-\text {e}^{-(n+1)k_v\varDelta t}}{1-\text {e}^{-k_v\varDelta t}}\text {e}^{-k_v (t - n\varDelta t)}, \end{aligned}$$

with

$$\begin{aligned} V(n\varDelta t)^+=V_0(1+\text {e}^{-k_v\varDelta t}+\cdots + \text {e}^{-nk_v\varDelta t}), \end{aligned}$$

where $V(n\varDelta t)^+$ is the level of vaccine just after $(n+1)$th boost, as the first boost is given at $t=0$. It is obvious that *V* tends to a periodic function as $t \rightarrow \infty$. More precisely,

6 $$\begin{aligned} V(t) \rightarrow V_0\frac{\text {e}^{-k_v \varDelta t \left( \tfrac{t}{\varDelta t} -\left[ \tfrac{t}{\varDelta t}\right] \right) }}{1-\text {e}^{-k_v\varDelta t}}=:V^{\infty }(t), \end{aligned}$$

where $s-[s]$ is the fractional part of $s=\tfrac{t}{\varDelta t}$. We see that $V(t) < V^{\infty }(t)$ for every $t>0$.

Then the asymptotic dynamics of $D_m$ is governed by

$$\begin{aligned} \dot{d}_m (t)=V(t)- k_m d_m, \end{aligned}$$

and as *V* is periodic, in the limit we also obtain a periodic expression for $d_m$:

$$\begin{aligned} d_m^{\infty }(t) = \frac{V_0}{(1-\text {e}^{-k_v})(1-\text {e}^{-k_m})} \frac{(1-\text {e}^{-k_m})\text {e}^{-k_v (t-[t])}- (1-\text {e}^{-k_v}) \text {e}^{-k_m(t-[t])} }{ k_m-k_v}, \end{aligned}$$

where we assume $\varDelta t =1$, for simplicity (one can always choose appropriate time units and scale all the parameters and variables accordingly).

Similarly, we can show that the functions $d_c(t)$, $d_r(t)$, *r*(*t*) and *c*(*t*) are asymptotically periodic with the period equal to 1. Clearly, asymptotic equation for any of these variables can be expressed in the following way

7 $$\begin{aligned} \dot{x} = F(t)-G(t) x, \quad x(0)=0, \end{aligned}$$

where *F* and *G* are periodic with the period 1, $G(t) > 0$ (in fact, for all the variables except *c* the function *G* is constant, $G(t)=k_{CR}$, or $G(t)=\mu _D$, or $G(t)=\mu _R$ for $d_c(t)$, $d_r(t)$, *r*(*t*), respectively).

### **Lemma 1**

For any solution *x*(*t*) of Eq. (7) there exists a periodic function $x^*$ with period 1 such that $|x(t)-x^*(t)|\rightarrow 0$ as $t \rightarrow \infty$.

### *Proof*

The solution of Eq. (7) reads

8 $$\begin{aligned} x(t)=\int \limits _0^t F(s)\text {e}^{-\int \limits _s^t G(u)du}ds. \end{aligned}$$

For $t=n\in \mathbb {N}$ Eq. (8) has the form

$$\begin{aligned} x(n)= \int \limits _0^n F(s)\text {e}^{-\int \limits _s^n G(u)du}ds = \sum \limits _{k=1}^n \text {e}^{-\int \limits _k^n G(u)du} \int \limits _{k-1}^k F(s) \text {e}^{-\int \limits _s^kG(u)du} ds. \end{aligned}$$

Due to periodicity of *G* we have

$$\begin{aligned} {\int \limits _k^n G(u)du} = (n-k) {\int \limits _0^1 G(u)du} = (n-k) G_A, \end{aligned}$$

where $G_A=\int _0^1 G(u)du$ is the mean value of *G*, while due to periodicity of *G* and *F* we obtain

$$\begin{aligned} \int \limits _{k-1}^k F(s) \text {e}^{-\int _s^kG(u)du} ds = \int \limits _{0}^1 F(s) \text {e}^{-\int \limits _s^1G(u)du} ds =: K. \end{aligned}$$

Therefore,

$$\begin{aligned} x(n)=K ( 1+ \text {e}^{-G_A}+\text {e}^{-2G_A}+\cdots + \text {e}^{-(n-1)G_A}) =\frac{1-\text {e}^{-nG_A}}{1-\text {e}^{-G_A}} K \end{aligned}$$

yielding

$$\begin{aligned} x(n) \rightarrow \frac{K}{1-\text {e}^{-G_A}}. \end{aligned}$$

Next, let us take $t\in (0,1)$ and calculate

$$\begin{aligned} \begin{aligned} x(n+t)&= \int \limits _0^{n+t} F(s)\text {e}^{-\int _s^{n+t} G(u)du}ds = \text {e}^{-\int _0^tG(u)du} \int \limits _0^n F(s) \text {e}^{-\int _s^n G(u)du} ds\\&\quad +\,\int \limits _n^{n+t}F(s)\text {e}^{-\int _s^{n+t} G(u)du}ds \\&=x(n) \text {e}^{-\int _0^tG(u)du} + \int \limits _0^t F(s)\text {e}^{-\int _s^{t} G(u)du}ds. \end{aligned} \end{aligned}$$

This proves that *x*(*t*) converges to the periodic function

$$\begin{aligned} x^*(t) = \frac{K}{1-\text {e}^{-G_A}}\text {e}^{-\int _0^{t-[t]}G(u)du} + \int \limits _0^{t-[t]} F(s)\text {e}^{-\int \nolimits _s^{t-[t]} G(u)du}ds. \end{aligned}$$

$$\square $$

Now, we need to study

9 $$\begin{aligned} \dot{x} = x \left( g - \frac{H(t)}{1+x} \right) , \quad x(t_0)= x_0, \end{aligned}$$

where $x=p$ and $H(t)=a c(t)$ is smooth and periodic. We can show that the dynamics of Eq. (9) depends on an average of *H*. Let us define $H_A=\int _0^1 H(s)ds$.

### **Theorem 3**

If $g > H_A$, then $x(t) \rightarrow \infty$, as $t\rightarrow +\infty$, for any $t_0$, and $x_0>0$. If $g<H_A$, then for any $t_0\in [0,1)$ there exists $x^*(t_0)>0$ such that

- for $0<x_0<x^*(t_0)$ the solution $x(t)\rightarrow 0$ as $t \rightarrow \infty$;
- for $x_0>x^*(t_0)$ the solution $x(t) \rightarrow \infty$ as $t \rightarrow \infty$;
- for $x_0=x^*(t_0)$ the solution is periodic.

### *Proof*

It is a simple corollary from Theorems 1 and 2.

### Conditions for cure and unsuccessful treatment

Now, we come back to Eq. (6), and consider together the magnitude of one boost $V_0$ with the frequency of vaccine application. For every $\varepsilon >0$ there exists $\bar{t}$ such that for any $t>\bar{t}$ we have

$$\begin{aligned} V \in \left[ V_0\frac{\text {e}^{-k_v \varDelta t}}{1-\text {e}^{-k_v \varDelta t}}-\varepsilon , V_0\frac{1}{1-\text {e}^{-k_v\varDelta t}} \right] =: [V_{m},V_{M} ]. \end{aligned}$$

Then, for sufficiently large *t* we obtain the following inequalities:

$$\begin{aligned} d_m \in \left[ \frac{V_{m}}{k_m}, \frac{V_{M}}{k_m} \right]=: & {} \left[ d_m^{m},d_m^{M}\right] ,\quad d_c \in \left[ \frac{d_m^{m}}{k_{CR}}, \frac{d_m^{M}}{k_{CR}}\right] =:[ d_c^{m},d_c^{M}], \\ d_r \in \left[ \frac{d_c^{m}}{\mu _D}, \frac{d_c^{M}}{\mu _D} \right]=: & {} \left[ d_r^{m},d_r^{M}\right] ,\quad r \in \left[ \frac{d_r^{m}}{\mu _R}, \frac{d_r^{M}}{\mu _R} \right] =:[ r_{m},r_{M}]. \end{aligned}$$

Hence, for sufficiently large *t*,

$$\begin{aligned} \dot{c} \ge d_c^{m}-\left( \mu _C+k r_{M} \right) c \quad \Longrightarrow \quad c \ge \frac{d_c^{m}}{\mu _C+k r_{M}}=: c_{m} \end{aligned}$$

and

$$\begin{aligned} \dot{c} \le d_c^{M}-\left( \mu _C+k r_{m} \right) c \quad \Longrightarrow \quad c \le \frac{d_c^{M}}{\mu _C+k r_{m}}=: c_{M} \end{aligned}$$

Eventually, *p* is governed by

$$\begin{aligned} g p - \frac{a c_{M} p}{1+p} \le \dot{p} \le g p - \frac{a c_{m} p}{1+p}, \end{aligned}$$

and therefore if $c_{m}>\frac{g}{a}$ and $p<\bar{p}_{\min } := \tfrac{a c_{m}}{g}-1$, then $p \rightarrow 0$ yielding the cure of the disease.

As $\varepsilon$ is arbitrary, we can take a limit $\varepsilon \rightarrow 0$ and calculating $c_{\min }$ we obtain

$$\begin{aligned} c_{\min }= \frac{\mu _R \mu _D V_{\min } }{k_m \mu _R \mu _D \mu _C k_{CR} +k V_{\max }}. \end{aligned}$$

Using the formula above we can approximate the value $V_0$ which is sufficient to cure the disease for the fixed interval $\varDelta t$, that is

$$\begin{aligned} \frac{\mu _R \mu _D \frac{V_0\text {e}^{-k_v\varDelta t }}{1-\text {e}^{-k_v\varDelta t}} }{k_m \mu _R \mu _D \mu _C k_{CR}+\frac{kV_0}{1-\text {e}^{-k_v\varDelta t}}}> \frac{g}{a} \end{aligned}$$

implying

$$\begin{aligned} \frac{V_0}{1-\text {e}^{-k_v \varDelta t }} (a \mu _R \mu _D \text {e}^{-k_v \varDelta t } -k g ) > g k_m k_{CR} \mu _C \mu _R \mu _D. \end{aligned}$$

This means that $g < g_{\max }$ is the necessary condition to obtain the cure independently of the type of treatment (one or more boosts).

#### **Corollary 3**

If

$$\begin{aligned} g < g_{\max {}}\text {e}^{-k_v \varDelta t}, \end{aligned}$$

then

$$\begin{aligned} V_0 > \frac{ g k_m k_{CR} \mu _C \mu _R \mu _D (1-\text {e}^{-k_v \varDelta t })}{k( g_{\max {}} \text {e}^{-k_v \varDelta t } -g) } \end{aligned}$$

is sufficient to cure the disease for $p_0<\bar{p}_{\min }=\tfrac{a c_{\min }}{g}-1$.

On the other hand, if $p_0>\bar{p}_{\max } =\tfrac{a c_{\max }}{g}-1$ or $c_{\max }<\frac{g}{a}$, then $p(t) \rightarrow \infty$ as $t \rightarrow \infty$.

At the end we should notice, that the result of Theorem 3 can be used for better approximation of the conditions for cure, but then the average value of *c* has to be calculated explicitly, which is possible but tedious task.

## Conclusion

The model proposed in Kronik et al. (2010) and analysed in the present work, describes the interaction between advanced PCa and immune system under vaccination treatment. The immune response is governed by a one-way signalling cascade starting from the tumour-specific antigen, which can be endogenous (represented by $V_p$) or externally administered (represented by *V*). The signal proceeds through antigen-presenting cells to the activation of cytotoxic effector cells (*c*). In parallel a built-in delayed negative feedback (carried out by *r*) inhibits results from the same stimulation, and inhibits the immune attack on the cancer. Without treatment, this system stabilises at certain level of effector cells ($c=c_{\infty }$), which contributes to the reduction of the tumour load. Corollary 1 gives the condition for a possibility of tumour control. In brief, these conditions demand that the pro-inflammatory arm of the system will be stronger than pro-regulatory arm and also be strong in comparison to the growth rate of the tumour. When this is the case, the outcome depends on the tumour load at the beginning of the process: if it is higher than a threshold $\bar{p}$, computed in Sect. 4, the disease will progress, while if it is lower than $\bar{p}$, disease will be eliminated.

One boost of vaccination has a transient effect on the system. A signal is sent from *V* to *c*, as described above, but eventually it wanes off, due to elimination in all the cell populations along the chain of reaction. Therefore, *c* temporarily increases above it’s steady level, $c_\infty$, but eventually goes back down to that level. It is reasonable to assume that at the beginning of the treatment the disease was not under control (i.e., $p>\bar{p}$, (otherwise the treatment would not be needed). Then, if the conditions of Corollary 1 hold, the boost $V_0$ should be high enough, so that the transient increase in *c* will reduce the tumour load *p* below the threshold $\bar{p}$. If this happens, the immune system may restore the control and eliminate the disease without additional treatments. If on the other hand, such substantial boost is unfeasible, or the parameters make it is impossible (violating assumptions of Corollary 1), a single vaccination cannot be effective against cancer. In particular this applies when, as assumed in Kogan et al. (2012) and Kronik et al. (2010), $V_p$ is zero or negligible.

In the latter case of ineffective endogenous antigen response, the hope is that periodic treatment may help. Indeed, as shown in Sect. 5, when the vaccination is applied periodically, the signal sent to the effector cells reaches higher levels, and can be practically evaluated to be higher than some minimal level $c > c_{\min } > c_{\infty }$. Thus, in principle a prolonged may generate an on-going immune reaction at levels higher than endogenous. As shown by Corollary 3, there is a possibility for elimination of tumour of any initial size, given that the treatment frequency ($\varDelta t$) and doses ($V_0$) can be controlled. Importantly, using this Corollary, or, if needed, more explicit numerical calculations, the minimal requirements of treatment intensity parameters (i.e., $\varDelta t$ and $V_0$) can be computed, given the values of the model parameters. This opens the path for optimisation of the immune treatment regimens, both on population and individual levels. We hope that our analysis will facilitate and motivate further advancement of mathematical and computational methods in the field of oncoimmunotherapy.
